# Prevalence of Painful Lesions of the Digits and Risk Factors Associated with Digital Dermatitis, Ulcers and White Line Disease on Swiss Cattle Farms

**DOI:** 10.3390/ani14010153

**Published:** 2024-01-02

**Authors:** Andreas Fürmann, Claudia Syring, Jens Becker, Analena Sarbach, Jim Weber, Maria Welham Ruiters, Adrian Steiner

**Affiliations:** Clinic for Ruminants, Department of Clinical Veterinary Medicine, Vetsuisse Faculty, University of Bern, 3012 Bern, Switzerland; claudia.syring@unibe.ch (C.S.); jens.becker@unibe.ch (J.B.); analena.sarbach@unibe.ch (A.S.); jim.weber@unibe.ch (J.W.); maria.welham@unibe.ch (M.W.R.); adrian.steiner@unibe.ch (A.S.)

**Keywords:** alarm lesion, claw disorders, cows, lameness, trimming

## Abstract

**Simple Summary:**

Lesions of the digits and associated lameness in cattle can not only cause pain and therefore impair animal welfare, but can also result in economic losses. Frequent claw trimming can prevent the development of claw disorders, but documentation of health data of the digits is essential for follow-up. These data are useful not only for farmers, veterinarians and herd health, but also for the establishment of claw health programmes on a regional basis. The objectives of this study were to present comparative prevalence data on painful lesions of the digits from over 700 cattle farms participating in a nationwide claw health programme in Switzerland over a three-year period. Furthermore, this study contributes to the identification of risk factors at the herd- and cow-levels regarding the occurrence of digital dermatitis, ulcers and white line disease in Swiss dairy cows. Factors found to be associated with these lesions may help to improve management factors contributing to better digit health on farms with small herds that have frequent access to pasture, as analysed in this study.

**Abstract:**

The first aim of this study was to calculate the prevalence of painful lesions of the digits (“alarm” lesions; ALs) in Swiss dairy herds and cow–calf operations over a three-year study period. The following ALs were included in the calculation: the M2 stage of digital dermatitis (DD M2), ulcers (U), white line fissures (WLF) of moderate and high severity, white line abscesses (WLA), interdigital phlegmon (IP) and swelling of the coronet and/or bulb (SW). Between February 2020 and February 2023, digit disorders were electronically recorded during routine trimmings by 40 specially trained hoof trimmers on Swiss cattle farms participating in the national claw health programme. The data set used consisted of over 35,000 observations from almost 25,000 cows from 702 herds. While at the herd-level, the predominant AL documented in 2022 was U with 50.3% followed by WLF with 38.1%, at the cow-level, in 2022, it was DD M2 with 5.4% followed by U with 3.7%. During the study period, within-herd prevalences of ALs ranged from 0.0% to a maximum of 66.1% in 2020. The second aim of this study was to determine herd- and cow-level risk factors associated with digital dermatitis (DD), U and white line disease (WL) in dairy cows using data from 2022. While for DD, analysed herd-level factors appeared to have a greater effect on the probability of its occurrence, the presence of U and WL was mainly associated with the analysed cow-level factors. The risk for DD increased with a higher herd trimming frequency. Herds kept in tie stalls had a lower risk for DD and WL and a higher risk for U compared to herds kept in loose housing systems. Herds with predominantly Holstein Friesian cows as well as Holstein Friesian cows had a higher risk for the occurrence of DD compared to herds and cows of other breeds. With increasing parity, cows had a higher risk of developing U and WL, whereas for DD, parity was negatively associated with prevalence. Cows trimmed during the grazing period had a higher risk of U and WL than cows trimmed during the housing period. These findings may contribute to improve management measures affecting the health of the digits in farms with structures similar to those evaluated in the current study, such as small herds with frequent access to pasture. Further research is warranted to demonstrate how measures addressing the current results combined with those of individual herd risk assessments might contribute to an improvement in the health of the digits in the respective dairy herds.

## 1. Introduction

Lesions of the digits and associated lameness have been receiving increasing amounts of attention in the literature on cattle farming worldwide [1,2,3]. The consequences of these disorders are manifold: they are the third-most-common cause of culling in dairy herds, along with having detrimental effects on udder health and fertility [1,4,5,6,7]. They result in significant economic losses due to reduced feed intake by the affected animals with a subsequent decline in the milk yield [1,8,9]. In addition, lameness impairs animal welfare [10] and can lead to an increase in greenhouse gas emissions due to reduced feed utilisation efficiency in lame cows [11,12].

To date, three studies have estimated the prevalence of disorders of the digits based on data from routine claw trimmings on Swiss cattle farms, where frequent occurrences were reported for heel–horn erosion (HHE), digital dermatitis (DD), white line disease (WL) and sole haemorrhage (SH) at both the herd and cow levels [1,7,13]. More specifically, these studies showed that the prevalence of DD increased from 5.3% to 20.7% at the cow level between 2002 and 2021 [1,7], that of sole ulcers increased from 0.4% to 4.2% between 2010 and 2021 [1,13], and that of WL increased from 4.8% to 16.3% over the same period [1,13]. High herd and cow prevalences of infectious claw diseases, particularly DD, and non-infectious claw diseases, particularly WL and ulcers (U), have also been reported in the international literature [3,10]. In a Canadian study, for example, it was found that 93.6% of the herds were affected by DD and 92.3% were affected by sole ulcers [3]. Lesions of the digits that are always associated with pain are referred to as “alarm lesions” (ALs) [14], and they frequently, but not always, result in detectable lameness [10].

To maintain the health of the digits, risk factors associated with digit disorders have been investigated in several countries [15,16,17,18,19]. Previous studies in Switzerland by Becker et al. (2014) and Bielfeldt et al. (2005) showed that herd and cow management principles, such as choice of housing type, breed and parity, as well as access to pasture, influence the occurrence of digit disorders [7,20]. However, these studies lack long-term follow-up and standardised data collection.

The project “Healthy claws–the foundation for the future” aims to establish regular claw health monitoring in Switzerland in close cooperation with professional hoof trimmers by electronically recording the findings on digit disorders during routine trimming [1,21,22]. The project is an innovation programme run by the Federal Office for Agriculture to promote the sustainable and efficient use of resources [23]. This includes the improvement of animal welfare through the sustainable management of farm animals, for which uniform collection, storage and evaluation of digit heath data is essential [23].

Based on the above, to effectively improve animal welfare, special attention should be paid to lameness-associated lesions with high prevalences such as DD, U and WL, for which an assessment of the associated risk factors is needed. The present study provides, for the first time, a large and up-to-date set of digit health data based on the electronic records of specially trained hoof trimmers. Although the literature on risk factors for individual digit disorders is extensive, we aimed to evaluate whether these risk factors are also applicable to small-sized dairy herds from mountainous regions.

The aims of the present study were (i) to determine the prevalence development of ALs on Swiss cattle farms based on an evaluation of electronical data recordings collected during routine herd trimmings for the years 2020 to 2023, and (ii) to assess the risk factors associated with DD, U and WL at both the herd- and cow-levels in dairy farms. We hypothesised that the implementation of the national claw health programme would lead to a decrease in the occurrence of ALs over time. Furthermore, we expected to identify risk factors referring to herd characteristics, claw health management and individual cow characteristics related to high prevalences of DD, U and WL.

## 2. Materials and Methods

This retrospective, longitudinal, observational study did not require ethical approval as no animal experiments were performed, but recorded data were analysed. Signed written informed consent was received from all farmers participating in this study, allowing the use of the production and health data of the involved cows for research purposes and publication thereof in anonymous form.

### 2.1. Study Area and Study Population

**Figure 1:**
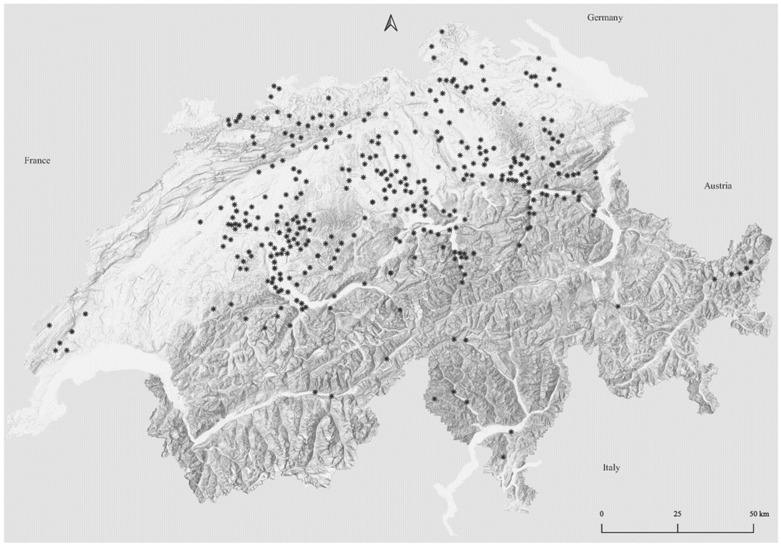
Map of Switzerland, depicting the locations of the enrolled farms using QGIS 3.12 (map: Swiss Confederation). As several farms may be located in a community with the same postal code, individual stars may represent more than one farm.

Cattle farms enrolled in this study were located in 24 of the 26 districts in Switzerland (Figure 1). Most farms were located in central Switzerland, while the French-speaking western and mountainous regions were under-represented. Since 2019, professional hoof trimmers have continuously been invited to participate in the government-initiated programme “Healthy claws–the foundation for the future”, which is headquartered at the Vetsuisse-Faculty of the University of Bern [1,21,24]. The study farms were recruited by the respective hoof trimmers joining the project or by farmers that contacted project members and were then assigned to a project hoof trimmer.

The prerequisite for participation was that the health data on the digits needed to be electronically documented by specially trained hoof trimmers according to the diagnoses and descriptions provided by the ICAR Claw Health Atlas and its Appendix 1 [25,26]. As the focus for the prevalence calculation was only on painful lesions of the digits, the following six ALs, as adapted by Kofler (2021), were considered [27]: (i) the M2 stage of digital dermatitis (DD M2); (ii) ulcers (U), including sole ulcers, bulb ulcers, toe ulcers and toe necrosis; (iii) white line abscesses (WLA); (iv) white line fissures (WLF) of moderate (two-thirds of the white line) and high severity (three-thirds of the white line); (v) swelling of the coronet and/or bulb (SW) and (vi) interdigital phlegmon (IP) [25,26]. Hoof trimmers were taught to classify WLF into three degrees of severity (one-third to three-thirds of the white line being affected), which is not described in the ICAR Claw Health Atlas but was used to calculate the prevalence of ALs [25,27].

The inclusion criteria were cattle holdings with dairy cows or cow–calf operations that underwent routine claw trimming at least once a year [1]. Only data from regular preventive trimmings were included. Data collected during therapeutic trimming visits or visits for lesion aftercare were excluded. To determine the lactation status, the first calving age of eligible cows had to be available. Consequently, farms that were not members of a breeding association and farms that kept the Eringer breed were excluded. According to Solano et al. (2016), a trimming session was defined as a single event if trimmings on the same farm were performed within a period of 15 days, as it sometimes takes more than one day to trim the whole herd [3]. Therefore, data from partial herd trimmings performed within these 15 days were allocated to the first trimming date of the respective session. If cows were trimmed twice in that period, the second observation was not considered. According to Solano et al. (2016), only whole herd trimmings, where ≥80% of cows were trimmed in one session, did not select cattle for lesion aftercare due to the lesion status [3]. Therefore, to minimise the selection bias in the prevalence estimation, only observations from whole herd trimmings were included in this study [3]. Furthermore, only data from one herd trimming per farm and year were included in the analysis. If data from more than one herd trimming within the same year were available, the value including the highest percentage of cows trimmed was considered. If the percentages of cows trimmed were identical, the trimming to be included was randomly selected with an R Studio script (R Core Team, 2018, Vienna, Austria; https://www.r-project.org/; accessed on 28 November 2023). Data originating from heifers (trimming date before the first calving date) were removed as the final step of data cleaning. Each farm as well as each cow finally appeared only once in the prevalence estimate of each year.

Data for the risk factor analyses consisted of a modified data set used for the prevalence estimation of ALs in 2022. Only data from dairy herds and dairy cows with complete milk records, as made available by breeding associations, were used. Analysed lesions of the digits for the risk factor analyses consisted of (i) all M stages of DD, as depicted and described in Appendix 1 of the ICAR Claw Health Atlas [26]; (ii) U, including sole ulcers, bulb ulcers, toe ulcers and toe necrosis and (iii) WL, including WLA and WLF, as depicted and described in the ICAR Claw Health Atlas [25]. In the risk factor analyses for DD, U and WL, 420 dairy herds and 13,735 dairy cows were included, as 77 cow–calf operations and 62 dairy herds were excluded due to missing milk recording data.

### 2.2. Electronical Data Recording

Participating hoof trimmers were each equipped with a tablet PC (Pokini Tab FS 12; EXTRA Computer GmbH, Giengen-Sachsenhausen, Germany), running the digit disorder documentation software “KLAUE” (version number 3.3.0.63; dsp-Agrosoft GmbH, Ketzin, Germany). The specific training programme used by these hoof trimmers was described by Strauss et al. (2021) [21]. In brief, participants initially received two days of group training from trained veterinarians at the faculty. This specialised training covered the use of the software and standardised diagnoses of digit disorders based on studying images depicted and described in the ICAR Claw Health Atlas and its Appendix 1 [25,26]. Hoof trimmers were then individually examined in terms of their disorder allocation and claw trimming techniques on farm. The test was completed successfully if agreement with the instructor was achieved for the disorder allocation based on images from the ICAR Claw Health Atlas and the examination of digit disorders during trimming with a kappa value ≥0.6 [21,24,25]. Only data from hoof trimmers who had successfully passed the examination were included in this study (n = 40). Hoof trimmers revealing kappa values <0.6 were retrained and retested (n = 7); data were used after successful retesting only. Data from hoof trimmers collected before they successfully completed training were excluded. All trimmers were subsequently offered annual mandatory advanced training courses focusing on disorder allocation, claw trimming techniques, recommendations for therapeutical measures and biosafety measures during claw trimming. Hoof trimmers received an allowance of SFr. 0.30 for each complete data set for a trimmed cow exported to the national claw health data storage platform “ClawNet” (Qualitas AG, Zug, Switzerland) [1].

When recording a herd, hoof trimmers collected specific herd data such as the housing system and stall characteristics using an electronical questionnaire stored in “KLAUE” software. For the collection of missing data for the risk factor analysis, farmers were contacted by telephone. Information concerning the production system and mountain pasturing were obtained from the National Livestock Register. Milk records and fertility data were provided by the breeding associations. Milk production data (parity, lactation length and milk yield) at the herd-level were given as annual averages in 2022, and at the cow-level, they were given as the most recent milk recording before the herd trimming date. The intercalving period was given as the annual average in 2022 at the herd-level and as the value of the last completed lactation at the cow-level.

### 2.3. Data Management

Health data for the digits were extracted from the platform “ClawNet”. For each cow, the presence of digit disorders was documented according to the ICAR Claw Health Atlas (1 = present vs. 0 = absent) at the cow-level, and these were then used to determine the prevalence at the herd-level [25]. Herd- and cow-level data were stored in two separate Excel sheets (Excel, Microsoft Office 2013, Microsoft Corp., Redmond, WA, USA). The proportion of cows affected with a certain lesion within a herd is referred to as the within-herd prevalence and was calculated in R. Trimming records from 2022 were divided into two periods—the grazing and housing periods—to analyse whether the occurrence of DD, U and WL was associated with changes in husbandry conditions. We hypothesised that an association between the environmental effect of the husbandry conditions in which the trimming occurred could be detected by assigning the trimming records to the appropriate period. The grazing season (1 May to 31 October) was defined by the RAUS programme. Participation in the federal government’s ethological programme “RAUS” (regular outdoor exercise) was voluntary [28]. From 1 May to 31 October, cows must be allowed to graze on pasture for at least 26 days per month [28]. From 1 November to 30 April, cows must be given access to an outdoor area or pasture for at least 13 days per month [28]. Therefore, the housing season was from 1 November to 30 April. A transition period of 60 days was included for U and WL, as described by Bergsten et al. (2015), because the effects of environmental changes on claw horn disruption lesions such as U and WL manifest themselves more slowly than skin diseases such as DD [29]. For DD, Bergsten et al. (2015) chose a shorter interval of 15 days [29]. For U and WL, trimmings during the grazing period included trimming records from 1 July to 31 December, whereas for DD, trimmings during the grazing period included trimming records from 16 May to 15 November. Trimming records gathered outside this period were considered to be trimmings during the housing period. As Holstein Friesian was the most commonly represented breed in the data set, the potential risk factor “breed” at the herd-level was categorised as either Holstein Friesian or other breed. The herd was allocated to the Holstein Friesian category if the largest proportion of dry and lactating cows in the respective herd was of that breed. 

### 2.4. Statistical Analyses

For statistical analyses, NCSS (https://www.ncss.com/; accessed on 28 November 2023) and R Studio software (R Core Team, 2018, Vienna, Austria; https://www.r-project.org/; accessed on 28 November 2023) were used. Categorical variables are described by frequency distributions, and continuous variables are summarised as the median and interquartile range. For the risk factor analyses, two separate analyses for the outcomes of interest were performed. The outcomes were (i) herds affected by DD, U or WL and (ii) cows affected by DD, U or WL. The outcomes were classified as binary (herd/cow affected vs. not affected), and each potential predictor variable was analysed with a respective univariable logistic regression model for each disease (i.e., three models each for the herd-level and cow-level). Only variables showing a *p*-value < 0.2 and that revealed no significant collinearity were included for further analysis. Model assumptions were checked before offering potential predictors to the model. Multicollinearity was checked using the mean square contingency coefficient phi. The linearity of the log odds of the outcomes and linear predictors was checked using Box–Tidwell tests. Subsequently, generalised linear models with the logit function were fitted to the data. Estimates were calculated as odds ratios with the respective 95% confidence interval (95% CI). To account for hierarchical ordered data, random effects for the farm and hoof trimmer were included. Controlling structures for mixed-model fitting were constructed for the WL model at the herd-level and DD and WL models at the cow-level through adding the “bobyqa” optimiser from the lme4 package. The best model fit was determined by eliminating the variable with the highest *p*-value when *p* > 0.05 and by a visual assessment of the residuals. We plotted the fitted against the residual values and evaluated the model through the use of the AIC. A stepwise backward elimination procedure was performed using the AIC to reach the best model fit. The level of significance for the models was set at *p* ≤ 0.05. Intra-class correlation coefficients (ICCs) were used for the description of error variance at the herd- and cow-levels.

## 3. Results

### 3.1. Herd and Cow Characteristics

**Figure 2:**
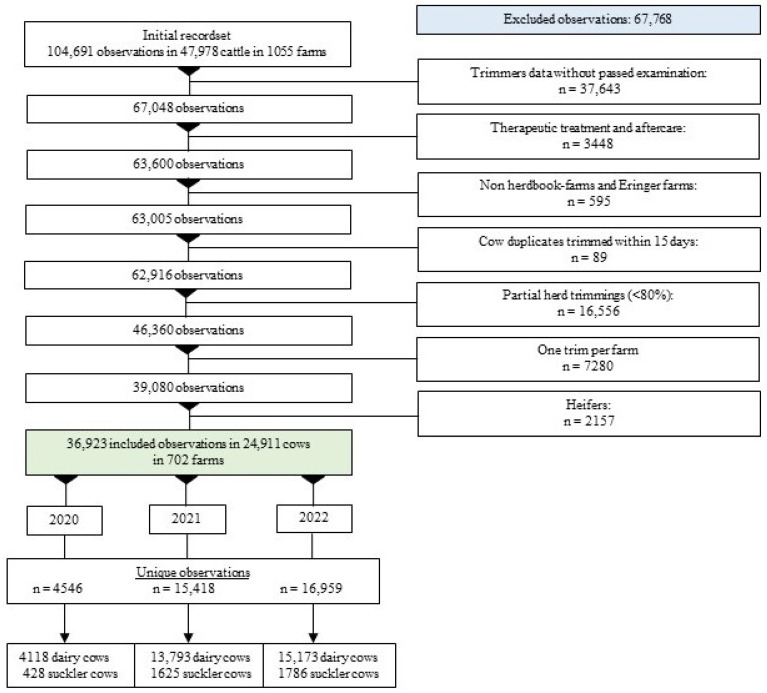
Flowchart showing the exclusion cascade of observations and the final allocation thereof to the years 2020 to 2022 for the calculation of the prevalence of alarm lesions of the digits in Swiss cows (n = number of cattle; suckler cows = cows in cow–calf operations).

A total of 36,923 observations from 24,911 cows from 702 farms recorded by 40 hoof trimmers (Figure 2) were included in the study, representing approximately 1.5% of farms and 3.5% of cows in Switzerland [30]. Table 1 and Table 2 show the characteristics of the herds and cows included over the three-year study period. The percentage distributions of both dairy herds and cow–calf operations, as well as the herd size and the proportion of the Holstein Friesian breed remained almost constant over the study period. Dairy herds predominated with 86.2% in 2022. The proportion of free stalls increased steadily over the study period, reaching 60.1% in 2022. Holstein Friesian cows predominated in one-third of the dairy herds. The other breeds included, among others, Swiss Brown, Swiss Fleckvieh and Simmental. The median herd size was 26 cows over all three study years. Table 3 presents milk production data for the dairy herds and cows evaluated in the risk factor analyses. Dairy cows had a median 305-day milk yield (kg) of 7702 in 2022 and were in their third lactation period (median) with a median milk yield of 25.8 kg before claw trimming. Due to the acquisition of newly participating farms during the study period, there were increases in the number of herds and cows included per hoof trimmer. The number of trimmings per year and herd also increased, since trimmings carried out in the first year of the study were electronically recorded mainly in the second half of the year, as many trimmers were still undergoing the initial training course in spring. Fewer hoof trimmers were documented for the last evaluated year because they dropped out of the project (n = 1) or did not submit data (n = 4). On some farms, herd trimmings were electronically recorded from the beginning of the study: 93 herds and 1885 cows appeared successively in all three study years, while 388 herds and 8723 cows were included in both the years 2021 and 2022.

### 3.2. Prevalence of Alarm Lesions

**Figure 3:**
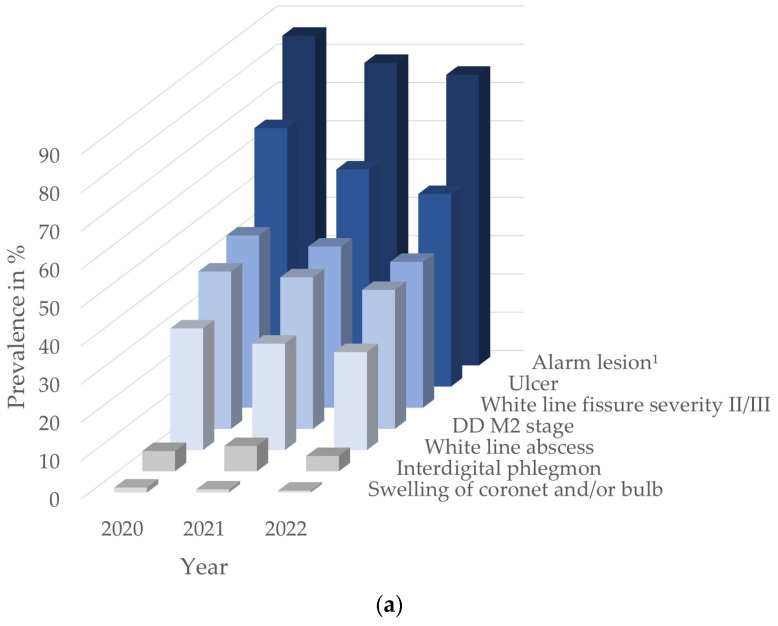

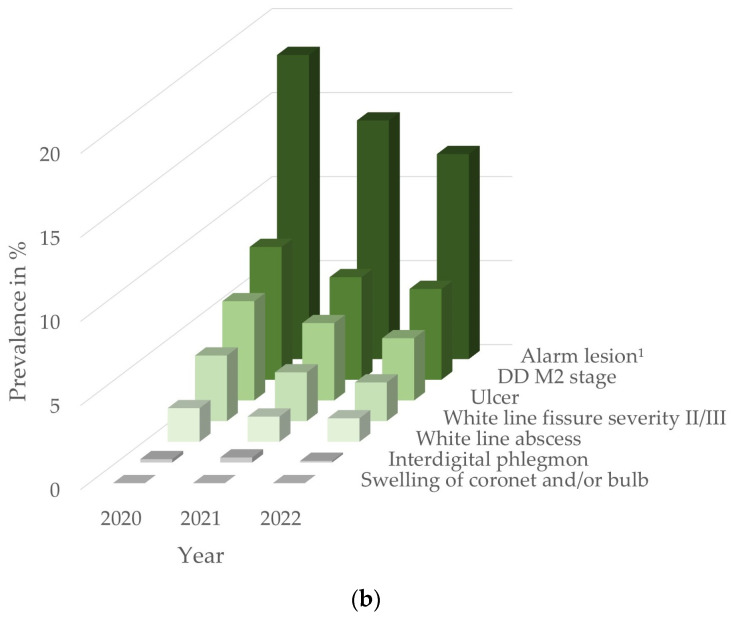
Graphical representation of the herd (**a**) and cow (**b**) prevalences of alarm lesions of the digits in Swiss cattle herds for the years 2020 to 2022. ^1^ Alarm lesion: (**a**) herds with at least one cow with an alarm lesion present and (**b**) cows with at least one alarm lesion present.

In total, 147,692 digits from 24,911 individual cows from 702 farms were examined Figure 3 graphically depicts the herd and cow prevalences of ALs over the three-year recording period.

Table 4 describes the herd, within-herd, and cow prevalences of ALs during the study period. At the herd-level, the predominant AL documented in 2022 was U with 50.3%, followed by WLF with 38.1% and DD M2 with 36.3%. Only for the ALs IP and SW was the prevalence at the herd-level in 2022 lower than 5%. Among U, sole ulcers with 44.0% and bulb ulcers with 13.1% in 2022 were most common. At the cow-level, DD M2 was the most prevalent AL with 5.4% in 2022, followed by U with 3.7% and WLF with 2.3%. For WLA, IP and SW, the median within-herd prevalence remained at 0.0% throughout the study period, while for DD M2, from 2020 to 2022, the median prevalence decreased from 4.1% to 0.0%, and for U, it decreased from 4.2% to 1.3%. The median percentage of cows per herd with an AL in 2022 was 8.0%. Only for U and WLF was the median within-herd prevalence above 0.0% in 2022. The percentage of herds with an AL present decreased by more than 10%, from 86.1% in 2020 to 75.9% in 2022. At the cow-level, the number of cows recorded with an AL decreased by almost 6% from 18.1% in 2020 to 12.2% in 2022. 

### 3.3. Risk Factors for Digital Dermatitis, Ulcers, and White Line Disease

In total, eight of the variables examined proved to be significant for the occurrence of at least one of the three analysed lesions in the univariable analyses at the herd- and cow-levels. The prevalences and results thereof are given in Tables S1 and S2. The results of the final multivariable regression models are presented in Table 5, Table 6 and Table 7. The ICCs ranged from 0.08 to 0.27 at the herd-level and from 0.19 to 0.31 at the cow-level.

#### 3.3.1. Herd-Level Risk Factors

In the multivariable model, significant herd-level risk factors for DD, U and WL in Swiss dairy herds were the number of herd trimmings per year, housing, mountain pasturing, predominant breed, intercalving period and lactation length.

The odds of a herd having DD increased by 64% per claw trimming, but the latter had no effect on U and WL. Tie stalls had 75% lower odds for having DD and 80% lower odds for the occurrence of WL as compared to free stalls. Likewise, farms that offered seasonal access to pastures at higher altitudes (i.e., mountain pasturing) had a 51% lower odds for the occurrence of DD. Furthermore, herds with predominatly Holstein Friesian cows had a five times higher odds of having DD than herds with a predominantce of other breeds. Production data also showed associations with the occurrence of DD and U. When the average intercalving period of a herd increased by one day, the odds of DD and U increased by 1%. The odds for U were 57% lower for herds with an average lactation length of less than 339 days than for those with an average lactation length of more than 360 days.

#### 3.3.2. Cow-Level Risk Factors

In the multivariable model, significant cow-level risk factors for DD, U and WL in Swiss dairy cows were housing, predominant breed, trimming season, parity and lactation length.

Cows housed in tie stalls had lower odds for DD and WL, while the odds for the occurrence of U were higher for cows kept in tie stalls. In addition, the odds for Holstein Friesian cows were 63% higher for DD, but 29% lower for WL. The trimming season showed significant associations with U and WL, with higher odds for trimmings during the grazing period (1 July 2022 to 31 December 2022). While the odds of DD decreased, they increased by 36% for U and by 30% for WL with each additional calving. Furthermore, the odds of WL were 19% lower for cows with a lactation length of less than 339 days, compared to cows with a lactation length of more than 360 days.

## 4. Discussion

Although ample research has been conducted to investigate the prevalences of different bovine digit disorders in Swiss cattle herds, information about painful lesions is missing. The prevalences from 2020 for the participating farms were assessed by Jury et al. (2021) in a previous study, but as they did not focus on ALs, the prevalences for that year were recalculated [1]. The present study contains the largest electronical data set on the health of the digits in Swiss cattle, with over 35,000 observations from almost 25,000 cows from over 700 farms. The implementation of stringent inclusion criteria ensured that we considered complete herd trimmings only (≥80% of cows per herd trimmed; median of trimmed cows per herd trimming = 100), where both lactating and dry cows were trimmed. Switzerland, with a median herd size of 16 dairy cows in 2001 [7], is gradually following the international trend of increasing the herd size and decreasing the number of dairy farms [30]. However, the median herd size of 26 cows found during the study period is still smaller when compared to the average herd size of over 50 dairy cows in the European Union [31], which makes it easier to trim a whole herd in one day compared to larger herds where partial herd trimmings are more common [32]. Allowing a small herd to be trimmed completely in a single day might have a positive effect on the accuracy of lesion recording [33]. The number of herds and cows trimmed varied among the study hoof trimmers. This may be due to a lack of full-time trimmers in Switzerland, as has already been described by Strauss et al. (2021) [21]. They observed that only seven out of thirty trimmers worked at least 90% of a full-time pensum [21]. We recognise that the amount of exported data from each trimmer is smaller than that of other studies using electronical trimming records. However, the exported data do not represent all farms that the trimmers work on, as only data from voluntarily participating farms with a valid written consent were exported to the data storage platform “ClawNet”. Using hoof trimmers data, Cohen’s kappa values of ≥0.6 for interobserver reliability are considered to have a substantial, but not excellent, strength of agreement [1,4,14]. A potential bias in prevalence estimates may be introduced due to the non-ideal sensitivity and specificity of this observer reliability test. As the disorders were assessed visually, there is also a potential risk for a misclassification bias [3]. In summary, Cohen’s kappa value is limited by the assumptions of rater independence and the lack of direct interpretability [34]. ICCs ranged from low to moderate. Random effects were therefore included in the model. High ICCs have already been reported by other studies, including those by Häggman and Juga (2015), Holzhauer et al. (2006) and Bielfeldt et al. (2005) [7,35,36]. This, once again, highlights the need for standardised training and further education of hoof trimmers in the recognition and documentation of lesions.

It has to be emphasised that the study population, representing 1.5% of Swiss cattle herds, is a subset of Swiss cattle herds, as there are no comparative electronic health data from farms that do not participate in the national claw health programme [30]. As we had to rely on the willingness of farmers to participate in this study and there was no randomised selection of study herds, a selection bias has to be considered [10]. Therefore, it is possible that both well-managed farms and those who aimed to improve digit health were over-represented in the data set used in this study [20].

Continuous claw trimming, recording, and providing farmers permanent access to their trimming data are important tools in the management and prevention of lame cows [37]. As currently only a small proportion of cattle trimmings in Switzerland is electronically recorded, the motivation for trimmers and farmers needs further attention. Documentation is not only needed by farmers to manage their herds or to monitor the occurrence of digit disorders, but also to propose benchmarks for the interpretation of health data on the digits [14,21,33]. Nevertheless, health data for the digits must be evaluated critically, and further investigation of disorder recordings by trimmers is needed to support the fact that recording routines do not deteriorate over time [33]. In general, the collection of trimming data in a standardised way allows the acquisition of large amounts of data sets, which are of high scientific value.

### 4.1. Prevalence Development for 2020–2023

The most common ALs were U at the herd-level and DD M2 at the cow-level. Within-herd prevalences of ALs ranged from 0.0% to a maximum of 66.1% in 2020, emphasising the need to improve farmers’ awareness of painful lesions and resulting lameness [37]. When comparing the prevalence data with those from other studies, it is important to keep in mind that not only herd management factors and climatic conditions but also training requirements and data collection methods differ. Therefore, the ICAR Claw Health Atlas was introduced to set international training standards and ensure the comparability of data [1,25,38]. However, reported lesion prevalences at the cow-level for DD M2 at 5.4%, U at 3.7% and WLA at 1.4% for 2022 in the current study were lower when compared to data from studies carried out in other countries. For instance, in Denmark, the prevalences of DD, U and WLA in cows were 21%, 6% and 3%, respectively [33]. In Canadian cows, including routine whole herd trimmings (≥80%), the prevalence of DD was 14.7%, while it was 5.7% for U and 4.4% for WL [3]. An Irish study also focusing on ALs (foul of the foot, hoof abscess, DD M2, sole ulcers, toe necrosis and WLA) showed a cow prevalence of 25% during the housing period with sole ulcers being the predominant AL with 12.7% [10].

Since hoof trimmers were not trained in lameness scoring, and because the software did not allow the insertion of lameness scores, these data are missing [1,32]. However, not all ALs result in detectable lameness [10,13]. In addition, lameness can be caused by disorders proximal to the digits [10,13]. A previous study conducted in Switzerland reported a lameness prevalence of 14.8% from 1449 cows on 78 farms assessed by veterinarian authors during routine trimmings, whereas in the present study, in 2022, 12.2% of all cows had an AL and might, therefore, show signs of lameness [13]. 

A lower prevalence of WLA compared to WLF was also found in a Danish study by Capion et al. (2021) [33]. This might be because the development of WLA can be prevented by the early treatment of WLF during trimming [33]. 

Two studies from Canada and Denmark also focused on long-term prevalence trends of lesions of the digits based on electronical trimming records [32,33]. In the Danish study, the prevalences of DD, U and WLA remained constant over the five-year study period [33]. In the Canadian study, the predicted mean herd prevalence of any type of lesion decreased from 45.1% in 2015 to 17.6% in 2018, with the prevalence of U decreasing from 17.7% to 3.1% during this period [32]. Over the three-year recording period used in the current study, the number of documented lesions decreased, suggesting that the digit health within the participating farms improved. One explanation for this finding might be that farmers, who joined the project voluntarily, were motivated to frequently perform routine trimming, lesion treatment and aftercare, which are known as efficient preventive measures for lameness [37]. Additionally, all participating farms were given the opportunity to receive an on-farm risk assessment from two project veterinarians free of cost, which was taken up by 113 of the participating farms (i.e., 16.1% of all evaluated farms). During these farm visits, the existing risk factors for the occurrence of digit disorders were discussed with the farmer and summarised in a written report. Subsequently, farmers were encouraged to implement targeted control measures on a voluntary basis. Further research is necessary to clarify whether these on-farm risk assessments may have influenced the trend for a decreasing prevalence over the study period. When assessing the observations of such studies, it should be mentioned that the documentation routine may have lost rigor over time. In order to overcome this potential source of observer bias in the present study, trimmers were invited to participate in a one-day free-of-cost annual refresher course where the recording software was updated and various training sessions focusing on lesion recording were held [39].

### 4.2. Risk Factors

#### 4.2.1. Digital Dermatitis

The more herd trimmings performed per year on a herd, the higher the odds of a herd being affected with DD. This finding is consistent with, among others, the study by Ahlén et al. (2022), who reported that farms with a trimming frequency of at least three times a year had a higher prevalence of DD than farms with a lower trimming frequency [40]. The contagious nature of this disease may have an impact, as *Treponema* spp. associated with DD can potentially be transmitted via claw trimming equipment [36,40]. Bayer et al. (2023) reported low implementation of external and internal biosafety measures during routine trimming in Swiss hoof trimmers, which could also explain our findings [41]. However, the advantages of frequent routine trimming outweigh the disadvantages, because the incidence of horn associated claw disorders can be reduced [37]. Nevertheless, the transmissibility of the infectious disease and the implementation of adequate biosafety measures must be considered [41]. Additionally, farms with higher DD prevalences may perform more frequent herd trimming sessions to prevent digit disorders, which accordingly leads to more frequent detection but also more treatment of such lesions [36,40].

Herds and cows kept in tie stalls had a lower risk of having DD. This finding agrees with studies by Weber et al. (2023), Ahlén et al. (2022) and Cramer et al. (2009) [24,40,42]. Tied cows tend to have improved lower leg hygiene compared to cows kept in free stalls, which are more exposed to slurry, a known risk factor for the occurrence of DD [24,43,44]. In addition, the spread of the pathogen may be more likely in loose housing systems than in herds kept in tie stalls [24].

Farms that allow their cattle to graze seasonally at high altitudes (i.e., mountain pasturing) had a lower risk of DD. This agrees with a recent study conducted in Switzerland by Weber et al. (2023) [24]. As the extensive keeping of cows on pastures in high altitudes in summer is specific to cattle management in mountainous areas, comparisons with other countries are difficult. Lower stocking densities on alpine pastures might prevent the distribution of DD, as it is mainly transmitted via skin-to-skin contact and faeces [24,45]. Most research reveals that farms offering their cows access to pasture are generally associated with a lower prevalence of DD [43,46]. In contrast, Holzhauer et al. (2006) found a positive association between >8 h access to pasture per day and the occurrence of DD [36,43]. Nevertheless, free access to pasture seems to not only be a protective factor for lameness in cows, but also promotes recovery from lameness [7,20,47].

Herds with predominantly Holstein Friesian cows as well as Holstein Friesian cows had higher odds for the occurrence of DD than “Non-Holstein Friesian” herds and cows. This was also found by Becker et al. (2014) and Holzhauer et al. (2006), who reported that the occurrence of DD is influenced by the angles of the dorsal wall [20,36]. These angles appear to be more acute in Holstein Friesian cows than in other breeds, causing lower bulbs and, therefore, tighter contact of the digit skin with the slurry [20,36]. Furthermore, Capion et al. (2021) reported that Holstein Friesian heifers had the highest prevalence of DD among crossbred, Jersey and Danish Red Dairy cows [33]. This finding underscores the need to quarantine newly purchased youngstock or reintroduced animals to prevent the introduction of DD [24].

With each additional calving, the risk of DD decreased at the cow-level. This is in line with studies by Capion et al. (2021), Gernand et al. (2012) and Somers et al. (2005), who reported that the risk of DD increased with lower parity, with the highest risk being attributed to primiparous cows [33,43,46,48]. This could be due to the physiological stress caused by metabolic and nutritional changes during and after the first calving, as well as the change in the housing system and stress associated stress with (re)integration into the herd [43,46,48]. Immunity may have further developed in multiparous cows that are exposed to the housing system for longer periods of time than younger cows [3,36,46,48]. Cows that reach a higher age may not be affected, while others may be culled at a younger age [43].

#### 4.2.2. Ulcers

Herds with an average lactation length of less than 339 days had lower odds of having U compared to herds with an average lactation length of more than 360 days. Charfeddine et al. (2017) reported that the occurrence of sole ulcers in early lactation was associated with more open days and a longer calving to first service interval [38]. Furthermore, the occurrence of severe sole ulcers in the first lactation period reduced the productive life (days from first calving to last milk record as the culling date, if the last milk records delivered exceeded 180 d) by up to 71 days [38]. This is in line with the findings of Olechnowicz and Jaskowski (2015) and Bicalho et al. (2007), who reported an extended range of 20 to 40 days from calving to conception [49,50]. Claw lesions such as U can affect ovarian activity through pain by increasing cortisol levels, which can interfere with the oestrus cycle and prolong the anoestrus period [38,51]. Furthermore, Häggman and Juga (2015), Liinamo et al. (2009) and Manske (2002) reported that the risk of U increases between 61 and 150 days of lactation, as lactational stress increases and negatively affects the body condition score [35,52,53]. It is also worth considering that many cows with a very long lactation period belong to the group of cows that are no longer inseminated and milked until being culled, so they are no longer prioritised for claw trimming by the farmer, which may then increase the risk of U.

Cows housed in tie stalls had a higher risk of suffering from U than cows kept in free stalls. For cows kept in tie stalls without outdoor access, Solano et al. (2016) found the opposite result [3]. However, comparability is impaired, as it is not as common for tied lactating cows to be kept at least part-time on pasture in Alberta, Canada as it is in Switzerland [3,54]. Tied Swiss cows are often kept on rubber mats covered with loose straw bedding. As mats age, the elasticity declines, resulting in a hard surface that may lead to increased pressure on the soles [55]. In addition, the stall length of tied cows is sometimes too short for their size, which causes the cows to stand with their hind legs on the rear edge of the curb, resulting in increased pressure on the soles [55]. Bernhard et al. (2020) reported that lame Swiss cows in tie stalls had a shorter lying time than their non-lame controls, which might further favour stress on the soles [56]. In addition, the restricted movement of tied cows negatively affects blood circulation and, therefore, the health of the digits [20]. Although the emphasis of dietary factors on the development of claw horn disruptions such as U appears to have diminished in recent decades through the use of ruminant-friendly total mixed rations, alimentary aspects might be involved. Common feeding techniques employed in tie stalls using a component-based diet, sometimes with an inadequate distribution of concentrates, may predispose cows to subacute rumen acidosis and increase the risk of developing U [55,57].

The risk of a cow presenting an U was the highest during the grazing period (defined for U as 1 July to 31 December). As described by Cook et al. (2004), environmental factors may explain the increase in claw lesions such as U [58]. Perhaps heat stress is one reason for changes in cow behaviours, leading to an increased standing time [35,52,59]. Additional mechanical stress on the bovine soles during the grazing period, caused by the daily movement from the stall to the pasture on sometimes stony trails, may be a contributing factor [35,52,60]. Our findings also are in line with those of Häggman and Juga (2015) and Bielfeldt et al. (2004), who showed that non-infectious claw disorders such as sole ulcers, sole haemorrhage, white line separation, chronic laminitis and double sole are mostly found during autumn trimming [7,35]. Holzhauer et al. (2008) explained this by the fact that, during the grazing period, the ration fed is not as stable as during winter feeding, which may favour the development of subacute rumen acidosis, which then may contribute to claw horn disruptions [61]. However, when interpreting these results, it is important to remember that cows had to access an outdoor area during the housing period [28].

The likelihood of a cow developing U increased with each additional calving, which was in accordance with several studies [3,35,37] and was explained by Sadiq et al. (2021) and Solano et al. (2016) in terms of a reduced horn quality due to prolonged exposure to hard flooring, potential trauma and metabolic changes around calving [3,37]. In multiparous cows, the risk of damage to the sole might further be promoted by a reduction in the thickness of the digital cushion and sinking of the pedal bone [7,35,37]. The increased probability of lesion recurrence in older cows also contributes to this finding [3,37,59].

#### 4.2.3. White Line Disease 

The only analysed herd-level risk factor that was significantly associated with the occurrence of WL was housing, which happened to also be significant at the cow-level. Herds and cows kept in tie stalls had a lower risk of suffering from WL compared to herds or cows kept in free stalls, a finding that was also reported by Häggman and Juga (2015) and Cramer et al. (2009) [35,42]. A Finnish study conducted by Häggman and Juga (2015) found a prevalence of non-infectious claw disorders (white line separations, sole haemorrhage, sole ulcers and chronic laminitis) of 41.9% in free stalls versus 26.2% in tie stalls [35]. In addition, Cramer et al. (2009) reported a zero prevalence for white line separations of 31.6% in free stalls versus 77.6% in tie stalls [42]. Prolonged standing, especially on hard surfaces, is known to impede blood flow in the lower limbs [32]. For example, prolonged waiting in the milking parlour in free stalls can lead to separation of the white line at the tip due to ischaemia and hypoxia [32,57,62]. Furthermore, overcrowding and the risk of slipping on smooth surfaces are known to be associated with WL [57,63]. We believe that certain barn construction characteristics in free stalls may influence the incidence of WL. Dead ends, excessively narrow aisles leading to tight turns, broken flooring elements and a widened scraper gutter may all stress the integrity of the white line [63]. It has also been reported that shear forces, e.g., from tight turns in the collecting yard, can be a cause of white line expansion [64].

Holstein Friesian cows had a lower probability of WL compared to other breeds. This outcome is not in line with several studies, as Holstein Friesians, as higher yielding cows, tend to show more non-infectious claw lesions than other breeds [20,35]. One reason for the latter finding is that Holstein Friesians carry more weight per unit area on their claws than other breeds and are, therefore, more susceptible to horn-associated claw disorders [65,66]. Additionally, they have a higher feed intake and are efficient at partitioning energy into milk, which favours the risk of subacute rumen acidosis when fed a high concentrate ration, which may contribute, in turn, to WL [58,65]. Bielfeldt et al. (2005) have already reported the lowest probability for WL in Swiss Holstein Friesian (odds = 0.8), the reason of which is not known, and we are currently not able to explain it [7]. 

## 5. Conclusions

The reported lesion prevalences highlight the importance of early detection and treatment of lameness to prevent the pain associated with these lesions for the improvement of animal welfare. Implementation of the national claw health programme led to a decrease in the occurrence of ALs over time. In our opinion, it seems to be crucial for farmers and involved veterinarians to have unrestricted and immediate access to trimming data in order to support the management of ALs to allow appropriate surveillance. Furthermore, the biosafety of Swiss cattle farms needs to be improved in order to reduce the prevalence of digital dermatitis. Primiparous Holstein Friesian cows kept in free stalls were revealed to have the highest odds for the occurrence of the infectious disease DD. Cows of higher parity housed in tie stalls had the highest risk for the occurrence of U. Breeds other than Holstein Friesian of higher parity kept in free stalls had the highest risk for the occurrence of WL. Considering the current results in combination with herd-specific risk assessments seems to represent a constructive basis for the implementation of measures with the goal to ameliorate the health of the digits of cows on small dairy farms that implement frequent access to pastures for their herds. Further studies are needed to evaluate this hypothesis.

## Figures and Tables

**Table 1:** Characteristics of the study herds included in the prevalence calculation of alarm lesions of the digits for the years 2020 to 2022.

Herd-Level Characteristic	Year 2020 ^1^ (151 Herds)		Year 2021 ^1^ (503 Herds)		Year 2022 ^1^ (559 Herds)	
	% (n)	Median (Q1–Q3) ^2^	% (n)	Median (Q1–Q3) ^2^	% (n)	Median (Q1–Q3) ^2^
Production system						
Milk	88.1 (133)		87.1 (438)		86.2 (482)	
Cow-calf operations	11.9 (18)		12.9 (65)		13.8 (77)	
Herd size ^3^		26 (16.5–40)		26 (18–40)		26 (18–39)
Housing type						
Tie stall	42.4 (64)		41.6 (209)		39.9 (223)	
Free stall	57.6 (87)		58.4 (294)		60.1 (336)	
Predominant breed (dairy herds)						
Holstein Friesian	33.1 (44)		32.2 (141)		33.4 (161)	
Other	66.9 (89)		67.8 (297)		66.6 (321)	
Herd trimmings per year ^4^						
n = 1	88.7 (134)		68.2 (343)		61.4 (343)	
n = 2	11.3 (17)		30.4 (153)		36.5 (204)	
n = 3	0.0 (0)		1.4 (7)		2.1 (12)	
Mountain pasturing Yes No	44.4 (67) 55.6 (84)		53.9 (271) 46.1 (232)		51.2 (286) 48.8 (273)	
Participating in the RAUS Programme ^5^	91.4 (138)		94.8 (477)		95.3 (533)	
Trimmed herds per hoof trimmer		8 (4–11.5)		10 (6–19)		10 (6–22)
Trimming season ^6^						
Housing season	40.4 (61)		65.0 (327)		69.4 (388)	
Grazing season	59.6 (90)		35.0 (176)		30.6 (171)	
Recording hoof trimmers	19		37		35	

^1^ 13 February to 12 February of the following year. ^2^ Q1 = 25th percentile; Q3 = 75th percentile. ^3^ Numbers of dry and lactating cows (trimming date after the first calving date). ^4^ ≥80% of the cows were trimmed. ^5^ Access to pasture (>26 days/month) from 1 May to 31 October; access to pasture or exercise in an outdoor pen (>13 days/month) from 1 November to 30 April. ^6^ Housing season = 1 November to 30 April; grazing season = 1 May to 31 October.

**Table 2:** Characteristics of the study cows included in the prevalence calculation of alarm lesions of the digits for the years 2020 to 2022.

Cow-Level Characteristic	Year 2020 ^1^ (4546 Cows)		Year 2021 ^1^ (15,418 Cows)		Year 2022 ^1^ (16,959 Cows)	
	% (n)	Median (Q1–Q3) ^2^	% (n)	Median (Q1–Q3) ^2^	% (n)	Median (Q1–Q3) ^2^
Production system						
Dairy cows	90.6 (4118)		89.5 (13,793)		89.5 (15,173)	
Suckler cows ^3^	9.4 (428)		10.5 (1625)		10.5 (1786)	
Percentage of trimmed cows per herd trimming ^4^		100 (95.7–100)		100 (94.1–100)		100 (95.5–100)
Trimmed cows per hoof trimmer		205 (135–309)		348 (135–573)		329 (182–639)

^1^ 13 February to 12 February of the following year. ^2^ Q1 = 25th percentile; Q3 = 75th percentile. ^3^ Cows in cow–calf operations. ^4^ ≥80% of the cows were trimmed.

**Table 3:** Milk records and fertility data for the dairy herds and cows evaluated in the final multilevel logistic regression model for the risk factor analyses for the occurrence of digital dermatitis, ulcers and white line disease.

	420 Dairy Herds and 13,735 Dairy Cows
Characteristic	Median (Q1–Q3) ^1^
Herd-Level ^2^	
305-day milk yield (kg)	7702 (6765–8668)
Parity	3 (2.6–3.4)
Lactation length (d)	346 (331–366)
Intercalving period	401 (385–418)
Cow-Level	
Milk yield (kg) ^3^	25.8 (20.2–32.3)
Parity ^3^	3 (2–4)
Lactation length (d) ^3^	316 (270–369)
Intercalving period (d) ^4^	388 (360–437)

^1^ Q1 = 25th percentile; Q3 = 75th percentile. ^2^ Mean value of the herd; assessed from 1 January 2022 to 31 December 2022. ^3^ Most recent milk recording before herd trimming. ^4^ Most recent lactation.

**Table 4:** Descriptive representation of the herd, within-herd, and cow prevalences of alarm lesions of the digits in the evaluated Swiss cattle herds for the years 2020 to 2023.

	Herd Prevalence			Within-Herd Prevalence									Cow Prevalence		
Year ^1^	2020	2021	2022	2020			2021			2022			2020	2021	2022
Total number analysed	151	503	559	151			503			559			4546	15,418	16,959
Data distribution statistic	%			Median	IQR ^2^	Range	Median	IQR ^2^	Range	Median	IQR ^2^	Range	%		
M2 stage of digital dermatitis	41.1	39.6	36.3	4.1	19.1	0–55.0	2.8	16.7	0–58.1	0.0	11.4	0–50.3	7.9	6.1	5.4
Interdigital phlegmon	5.3	6.6	4.0	0.0	0.0	0–11.8	0.0	0.0	0–18.2	0.0	0.0	0–9.1	0.2	0.3	0.1
Swelling of coronet and/or bulb	1.3	0.8	0.5	0.0	0.0	0–3.6	0.0	0.0	0–1.7	0.0	0.0	0–4.2	0.0	0.0	0.0
Ulcers ^3^	67.5	56.7	50.3	4.2	9.0	0–50.0	2.9	6.5	0–30.8	1.3	5.4	0–35.9	5.9	4.6	3.7
Sole ulcer	55.0	48.1	44.0	2.7	6.5	0–25.0	0.0	5.3	0–30.8	0.0	4.2	0–33.3	2.6	3.5	2.9
Bulb ulcer	19.9	15.7	13.1	0.0	0.0	0–50.0	0.0	0.0	0–25.0	0.0	0.0	0–18.8	0.9	0.7	0.5
Toe ulcer	10.6	4.6	4.5	0.0	0.0	0–5.0	0.0	0.0	0–14.1	0.0	0.0	0–5.6	0.5	0.2	0.2
Toe necrosis	5.3	5.4	4.5	0.0	0.0	0–5.0	0.0	0.0	0–5.3	0.0	0.0	0–6.3	0.2	0.2	0.2
White line fissure severity II/III	45.0	42.1	38.1	6.8	19.1	0–47.2	7.2	13.9	0–39.2	5.5	13.2	0–35.1	3.9	2.9	2.3
White line abscess	31.8	27.8	25.6	0.0	2.5	0–25.7	0.0	1.7	0–28.6	0.0	0.6	0–22.2	2.0	1.5	1.4
Alarm lesion ^4^	86.1	79.0	75.9	11.5	16.6	0–66.1	9.4	12.8	0–57.2	8.0	13.2	0–53.7	18.1	14.2	12.2

^1^ 13 February to 12 February of the following year. ^2^ IQR = interquartile range. ^3^ Distribution of different types of ulcers in the herds and cows analysed. In some herds and cows, more than one type of ulcer can occur. ^4^ Herds and cows with at least one alarm lesion present at routine trimming. Alarm lesions analysed include all lesions listed in the table.

**Table 5:** Final multilevel logistic regression model of herd- and cow-level factors associated with the occurrence of digital dermatitis in 13,735 dairy cows from 420 Swiss dairy farms participating in the national claw health programme in 2022.

Predictor	Digital Dermatitis					
	Class	Count (n)	Diseased (n)	Odds Ratio	95% CI ^1^	*p*-Value
Herd-Level						
Housing	Tie stall	194	62	0.25	0.15–0.41	<0.001
	Free stall	226	147	Ref		
Mountain pasturing	Yes	221	88	0.49	0.31–0.77	0.002
	No	199	121	Ref		
Predominant breed	Holstein Friesian	125	97	5.03	3.00–8.66	<0.001
	Other	295	112	Ref		
Herd trimmings per year	^2^			1.64	1.03–2.64	0.039
Intercalving period (d)	^2^			1.01	1.00–1.02	0.005
Cow-Level						
Housing	Tie stall	4228	170	0.44	0.31–0.63	<0.001
	Free stall	9507	1442	Ref		
Breed	Holstein Friesian	6072	1112	1.63	1.37–1.94	<0.001
	Other	7663	500	Ref		
Parity	^2^			0.97	0.94–1.00	0.046

^1^ 95% confidence interval. ^2^ Metric variable.

**Table 6:** Final multilevel logistic regression model of herd-and cow-level factors associated with the occurrence of ulcers in 13,735 dairy cows from 420 Swiss dairy farms participating in the national claw health programme in 2022.

Predictor	Ulcers					
	Class	Count (n)	Diseased (n)	Odds ratio	95% CI ^1^	*p*-Value
Herd-Level						
Intercalving period (d)	^2^			1.01	1.00–1.02	0.012
Lactation length (d)	<339	175	66	0.43	0.25–0.76	0.003
	340–360	118	67	0.79	0.45–1.40	0.419
	>360	127	85	Ref		
Cow-Level						
Housing	Tie stall	4228	180	1.47	1.11–1.96	0.008
	Free stall	9507	332	Ref		
Trimming season ^3^	Housing period	9022	200	0.65	0.50–0.85	0.002
	Grazing period	4713	312	Ref		
Parity	^2^			1.36	1.31–1.41	<0.001

^1^ 95% confidence interval. ^2^ Metric variable. ^3^ Housing period: 1 January to 30 June (60-day transition period included); grazing period: 1 July to 31 December (60-day transition period included).

**Table 7:** Final multilevel logistic regression model of herd- and cow-level factors associated with the occurrence of white line disease in 13,735 dairy cows from 420 Swiss dairy farms participating in the national claw health programme in 2022.

Predictor	White Line Disease					
	Class	Count (n)	Diseased (n)	Odds Ratio	95% CI ^1^	*p*-Value
Herd-Level						
Housing	Tie stall	194	115	0.20	0.11–0.34	<0.001
	Free stall	226	197	Ref		
Cow-Level						
Housing	Tie stall	4228	278	0.40	0.32–0.50	<0.001
	Free stall	9507	1199	Ref		
Breed	Holstein Friesian	6072	573	0.71	0.61–0.84	<0.001
	Other	7663	904	Ref		
Trimming season ^2^	Housing period	9022	596	0.55	0.44–0.67	<0.001
	Grazing period	4713	881	Ref		
Lactation length (d)	<339	4140	438	0.81	0.69–0.95	0.008
	340–360	5731	584	0.89	0.78–1.03	0.119
	>360	3864	453	Ref		
Parity	^3^			1.30	1.27–1.33	<0.001

^1^ 95% confidence interval. ^2^ Housing period: 1 January to 30 June (60-day transition period included); grazing period: 1 July to 31 December (60-day transition period included). ^3^ Metric variable.

## Data Availability

Data are contained within the article.

## Supplementary Materials

The following supporting information can be downloaded at: https://www.mdpi.com/article/10.3390/ani14010153/s1, Table S1: Prevalences and distribution of categorical herd-level variables for digital dermatitis, ulcers and white line disease in 420 Swiss dairy farms participating in the Swiss claw health programme in 2022 determined by univariable analyses; Table S2: Prevalences and distribution of categorical cow-level variables for digital dermatitis, ulcers and white line disease in in 13,735 dairy cows from 420 Swiss dairy farms participating in the Swiss claw health programme in 2022 determined by univariable analyses.
