# Supplementary material for: Prevalence of Painful Lesions of the Digits and Risk Factors Associated with Digital Dermatitis, Ulcers and White Line Disease on Swiss Cattle Farms
Source: Animals (Basel). 2024 Jan 2;14(1):153. doi: 10.3390/ani14010153 (PMC10778466; doi:10.3390/ani14010153)
Supplement: Supplementary file 1 [file animals-14-00153-s001.zip › animals-2742672-supplementary.pdf]

Table S1. Prevalences and distribution of categorical herd-level variables for digital dermatitis, ulcers and white line disease in 420 Swiss dairy farms participating in the Swiss claw health programme in 2022 offered to univariable analyses.

	**Digital Dermatitis**		**Ulcer**		**White Line Disease**	
	% (n)	P-value	% (n)	P-value	% (n)	P-value
Prevalence at herd-level in % (N)	49.8 (209)		51.9 (218)		74.3 (312)	
Variable						
Herd trimmings per year		<0.001		0.025		0.395
n = 1 (N = 247)	42.5 (105)		46.6 (115)		74.1 (183)	
n = 2 (N = 166)	59.6 (99)		59.0 (98)		75.3 (125)	
n = 3 (N = 7)	71.4 (5)		71.4 (5)		57.1 (4)	
Housing		<0.001		0.359		<0.001
Tie stall (N = 194)	32.0 (62)		46.4 (90)		59.3 (115)	
Free stall (N = 226)	65.0 (147)		56.6 (128)		87.2 (197)	
Mountain pasturing		<0.001		0.134		0.016
Yes (N = 221)	39.8 (88)		48.4 (107)		69.7 (154)	
No (N = 199)	60.8 (121)		55.8 (111)		79.4 (158)	
Predominant breed		<0.001		0.016		0.647
Holstein Friesian (N = 125)	77.6 (97)		59.2 (74)		75.2 (94)	
Other (N = 295)	38.0 (112)		48.8 (144)		73.9 (218)	
Mean 305-d milk yield (kg)		<0.001		0.011		0.436
≤7000 (N = 134)	32.8 (44)		47.0 (63)		72.4 (97)	
7001-9000 (N = 218)	51.4 (112)		51.8 (113)		73.9 (161)	
>9000 (N = 68)	78.0 (53)		61.8 (42)		79.4 (54)	
Intercalving period (d)		0.006		<0.001		0.561
≤390 (N = 145)	36.0 (63)		37.7 (66)		73.1 (128)	
391-419 (N = 175)	61.3 (73)		56.3 (67)		75.6 (90)	
≥420 (N = 100)	57.5 (73)		66.9 (85)		74.8(95)	
Lactation length (d)		0.004		<0.001		0.587
≤339 (N = 175)	36.0 (63)		37.7 (66)		73.1 (128)	
340-360 (N = 118)	61.3 (73)		56.3 (67)		75.6 (90)	
≥361 (N = 127)	57.5 (73)		66.9 (85)		74.8(95)	

Table S2. Prevalences and distribution of categorical cow-level variables for digital dermatitis, ulcers and white line disease in in 13,735 dairy cows from 420 Swiss dairy farms participating in the Swiss claw health programme in 2022 offered to univariable analyses.

	**Digital Dermatitis**		**Ulcer**		**White Line Disease**	
	% (n)	P-value	% (n)	P-value	% (n)	P-value
Prevalence at cow-level in % (N)	11.7 (1,612)		3.7 (512)		10.8 (1,477)	
Variable						
Housing		<0.001		0.040		<0.001
Tie stall (N = 4,228)	4.0 (170)		4.3 (180)		6.6 (278)	
Free stall (N = 9,507)	15.2 (1,442)		3.5 (332)		12.6 (1,199)	
Predominant breed		<0.001		0.305		<0.001
Holstein Friesian (N = 6,072)	18.3 (1,112)		4.1 (251)		9.4 (573)	
Other (N = 7,663)	6.5 (500)		3.4 (261)		11.8 (904)	
Trimming season[1]		0.008		0.008		<0.001
Housing period (N = 9,022)	11.0 (970)		2.8 (200)		8.5 (596)	
Grazing period (N = 4,713)	13.0 (642)		4.7 (312)		13.2 (881)	
Milk yield (kg)						
≤20 (N = 3,393)	8.9 (301)	0.195	3.2 (109)	0.011	10.3 (351)	0.080
21-30 (N = 5,996)	10.9 (655)	0.139	3.7 (220)	0.141	10.4 (622)	0.054
≥31 (N = 4,346)	15.1 (656)		4.2 (183)		11.6 (504)	
Parity		0.011		<0.001		<0.001
n = 1 (N = 3,347)	10.1 (337)		1.2 (40)		5.8 (194)	
n = 2-3 (N = 5,614)	13.9 (780)		2.5 (141)		8.3 (468)	
n = ≥4 (N = 4,774)	10.3 (495)		6.9 (331)		17.1 (815)	
Intercalving period (d)						
≤365 (N = 4,388)	10.7 (470)	0.049	3.4 (150)	<0.001	10.8 (474)	<0.001
366-414 (N = 4,945)	12.2 (603)	0.177	4.3 (213)	0.027	12.3 (608)	0.073
≥415 (N = 4,402)	13.6 (599)		5.8 (257)		13.8 (609)	
Lactation length (d)						
≤339 (N = 4,140)	9.9 (409)	0.039	3.7 (151)	0.221	10.6 (438)	0.038
340-360 (N = 5,731)	11.4 (657)	0.767	3.3 (189)	0.031	10.2 (584)	0.026
≥361 (N = 3,864)	14.0 (541)		4.5 (172)		11.7 (453)	

[1]Grazing period: For DD = May 16 to Nov 15; For U and WL = Jul 01 to Dec 31.
Housing period: For DD = Nov 16 to May 15; For U and WL = Jan 01 to Jun 30.
